# Gout

**Published:** 1899-07

**Authors:** Jay F. Schamberg

**Affiliations:** Philadelphia


### GOUT.[1]

BY JAY F. SCHAMBERG, A.B., M.D., PHILADELPHIA.

The modern term gout was first employed in the thirteenth century by Radulphus or Rudolph. It is derived from the Latin word *gutta*, which means drop, for it was supposed that the peccant humors that gave rise to gout filtered drop by drop into the joints. The French have maintained the identity of these two words, so that *la goutte* means both gout and a drop.

Gout is a disease that traces its origin far back to the beginning of history. We read in the Bible that Asa, the great-grandson of Solomon, who reigned nine hundred years before the present era, was a victim of this malady. The twenty-third verse of the fifteenth chapter of I. Kings says, "Nevertheless in the time of his old age he was diseased in his feet;" and later, in II. Chronicles, xvi. 12, "And Asa in the thirty and ninth year of his reign was diseased in his feet, until his disease was exceeding great: yet in his disease he sought not to the Lord, but to the physicians." It is evident that the latter were not able to greatly prolong his life, for the following verse reads, "And Asa slept with his fathers, and died in the one and fortieth year of his reign."

The indolence, licentiousness, and intemperance that occurred at the time of the Roman decadence were favorable factors for the development of this disease. Seneca complains of the luxury that debauched the imperial Romans, so that even the ladies of the court were bald and fat and gouty as a result of their earnest devotion to Venus and Bacchus. From the beginning of English history to the present day there are few prominent men of Great Britain who have not suffered from the pangs of this great arch-enemy of luxury. Even the illustrious Sydenham, whose sound observations did much to clarify the obscurity that had surrounded this disease, had in his own person excellent clinical material for study. Indeed, he ultimately died of an acute attack of gout. And in recent years the valuable researches of Haig were primarily undertaken in a tedious but finally successful endeavor to rid himself of one-sided headaches to which he had been subject for years, and which he discovered to be of gouty origin.

A tremendous amount of literature has been written about the

---

[1] Read before the Academy of Stomatology, March 28, 1899.

nature and ultimate causes of gout, and yet at the present time there are many points in its chemistry and pathology upon which it is hazardous to dogmatize. There is much conflicting evidence at hand, the fruit of years of labor on the part of medical scientists. There is, however, one fundamental fact upon which all are in accord,—namely, that gout is the result of an excessive amount of the salts of uric acid in the blood. This has been as indubitably proven as any fact in medicine. The origin of uric acid and the cause of its precipitation in the tissues are points which have not been so satisfactorily settled.

1. Garrod claims that an acute attack of gout is invariably produced by an excess of uric acid in the blood, due to an increased formation and to a greatly decreased elimination. Also that the inflammation is caused by the deposition in the joints of urate of sodium.

2. Haig contends that there is a diminished alkalinity of the blood, and that the latter cannot therefore hold the uric acid in solution, so that it is deposited in the form of urates. He dissents from the view that an excess of uric acid is formed.

3. Ebstein presupposes a necrotic condition of the cartilages of joints due to the presence of dissolved urates, and believes that this invites the deposition of crystals of urates from a blood surcharged with uric acid.

4. The recent experiments of Luff have thrown considerable light upon the subject. In the Goulstonian lectures, delivered before the Royal College of Physicians of London, he formulated the following conclusions:

1. Uric acid is not normally present in the blood of man.

2. Uric acid is normally produced only in the kidneys.

3. Uric acid is normally formed from urea, probably by a conjugation of that substance with glycocin in the kidneys.

4. Uric acid is present in the blood in gout as the soluble sodium quadrurate. It is deposited from the blood as the sodium biurate.

5. The presence of uric acid in the blood is due to deficient excretion by the kidneys, and to subsequent absorption from the kidneys into the blood.

6. Gout is probably always preceded by some affection of the kidneys, functional or organic, which interferes with the proper excretion of uric acid. The probable seat of the kidney affection giving rise to gout is in the epithelium of the convoluted tubules.

7. In certain blood-diseases and disorders, accompanied by leucocytosis, uric acid is formed within the system from nuclein. In such circumstances it passes at once into the blood and is rapidly eliminated by the kidneys.

Luff's experiments controvert certain long-cherished theories concerning the chemistry of gout. It has heretofore been commonly asserted that the production of uric acid was an intermediate stage in the final formation of urea. If Luff's conclusions are true, then the reverse is the case,—namely, that uric acid is an ultimate product formed from the union of urea and glycocin, which is a substance elaborated in the liver. This view had been previously maintained by Sir Alfred Garrod and Sir William Roberts. These gentlemen, furthermore, believed that the accumulation of uric acid in the blood of gouty patients is due to deficient excretion rather than to increased production. Sir Alfred Garrod holds the view that among the causes exciting a gouty fit is a functional failure on the part of the kidneys. In chronic gout this functional failure is followed by structural changes in the kidneys.

The primary pathological cause of gout then is, according to Garrod, Roberts, and Luff, a defective capacity of the kidneys for the elimination of uric acid. Levison states that gout cannot be developed unless a primary renal lesion is present, and that this is almost invariably of the nature of an interstitial change.

In what manner does uric acid produce the symptoms of gout? The probabilities are that the various symptoms are directly attributable to the mechanical irritation produced by the deposition of crystals of the biurate of sodium. In the case of articular gout, the crystals present in the cartilages of joints give rise to inflammation and subsequently to degenerative changes. In the case of visceral gout, there is good reason to believe that the various symptoms are in a large measure due also to the mechanical effect of the uratic shower. The mere presence of uric acid in the blood does not seem to injuriously affect the organism. Immediately preceding an attack of gout, the patient usually enjoys excellent health. Again, in leukæmia and other diseases in which leucocytosis is present, uric acid is found in the blood, but it is rapidly eliminated and does no harm. It is its precipitation in the tissues and organs that is productive of the morbid phenomena of the disease.

After death we find that the cartilages and synovial membranes of joints are infiltrated with a chalky material which is fluid in its earlier state, but which later contains crystalline masses. The periosteum is frequently infiltrated with urates, but the bones, although they may be extensively diseased, remain free from the deposition. Sometimes post-mortem examination reveals uratic deposit in joints that had never given clinical evidences of this condition during the life of the patient. Uratic deposits are frequently found, in addition, in bursæ and in the subcutaneous connective tissue. They have also been discovered in the valves of the heart, the cartilages of the larynx, in the liver, kidneys, brain, and other organs.

*Etiology.*—Among the various factors that give rise to gout, heredity occupies a position of prominence. Garrod states that "more than one-half of all gouty patients can distinctly trace their ailment to an inherited taint." Paternal transmission of the disease is much more common than perpetuation through the mother.

Articular gout is overwhelmingly more frequent in men than in women. Out of six hundred and forty-eight cases reported, but thirty-two were females.

The character and quantity of food are factors of the greatest importance. Excesses in both eating and drinking may be set down as among the most potent of all of the causes of gout. The nitrogenized foods are those that are most harmful, the chief offender, of course, being meat. It is popularly believed that butcher's meat is much more injurious than the flesh of fowl. This is erroneous. Butcher's meat contains about nineteen per cent. of albuminoid matter, whereas fowl contains twenty per cent. and game twenty-two per cent. Fish comes next with seventeen per cent.; eggs, thirteen per cent.; oatmeal, twelve per cent.; bread, eight per cent.; rice and green peas, six per cent. Milk contains but four per cent., and green vegetables and fruits from one-half to two per cent. The amount of food ingested is an important element to be taken into consideration. It is evident that one might partake of the most highly nitrogenized foods provided the quantities were sufficiently small. For instance, cheese contains thirty per cent. of albuminoid matter, yet in the quantity usually eaten it is not potent for harm. Haig says that any one who indulges in meat twice a day, and in addition partakes of wine or malted liquor, may confidently expect to be gouty when he reaches the age of forty or forty-five.

From the remotest antiquity the deleterious influence of wine and other alcoholic beverages has been commented upon. It is not by virtue of the alcohol contained in these drinks, nor even because of their acidity, that their baneful effect is exerted. The gout inducing properties of wines seem to be dependent upon the effect they exert upon liver metabolism and consequently upon the production of glycocin. The heavier wines, such as port, sherry, and Madeira, head the list of gout-determining wines. Considerably less injurious are Burgundy and Champagne. Least detrimental are claret, hock, and Moselle. The distilled liquors, such as brandy and whiskey, have comparatively little gout-inducing influence, although their alcoholic percentage is, of course, extremely high. It is not alcoholism but gluttony that calls gout into existence. For this reason the inordinate use of the malt liquors is particularly apt to give rise to gout. The moderate price of beer, porter, and ale enables the masses to use them in extraordinary quantities. The watermen who raise ballast from the Thames are frequently afflicted with what is called "poor man's gout," which is due to the prodigious amounts of malt of which they dispose. Budd states that twelve quarts of porter in a day is not an uncommon average. Those of you who have sojourned in small university towns in Germany are familiar with the enormous beer-drinking capacity of the German students. To imbibe fifty or sixty "krüge" during the course of an evening is but an achievement of mediocrity. In whiskey-drinking Scotland and potato-eating Ireland gout is rare as compared with malt-consuming England and Germany. In the United States gout occurs almost solely among the rich and luxury-loving element of the population. Another not infrequent etiologic factor in the production of gout is lead-poisoning. Garrod found that thirty per cent. of the hospital cases had been painters or workers in lead. Not improbably it is through the injurious effect of lead on the kidneys that gout is thus produced.

*Symptomatology.*—When one glances over the long array of symptoms that gout is convicted of producing, one is apt to remark, "Verily, thou hast enumerated all the diseases to which flesh is heir." And yet this interminable list is no exaggeration. It is not necessary to have one's big toe swathed in bandages nor one's fingers embellished with chalk-stones in order to admit the existence of gout. It has subtler manifestations which are capable of causing the diagnostician much difficulty. The classic attack of gout is familiar to all. The patient, after experiencing prodromal cramp-like contractions of the calf, is suddenly seized, usually in the early hours of the morning, with violent pain in the metatarso-phalangeal articulation of the great toe, which subsequently becomes reddened, hot, shining, and swollen. The body temperature rises to 102° or 103° F. After a few hours of intolerable suffering, there occurs an abatement of the pain and fever, the patient usually lapsing into a peaceful slumber. During the day the patient is apt to feel easier, but with the oncoming of the night there occurs an aggravation of the pain, until the total subsidence of the attack at the end of several days or a week. These so-called fits of gout recur from time to time, the intervals of freedom varying according to the mode of living of the patient. After several such attacks the disease arrives at its chronic stage. Other joints than the great toe then become involved. Their order of frequency is as follows: the heels, the small joints of the hands, the ankles and wrists, the knees and elbows, and, finally and rarely, the hips and shoulders. These joints are apt to become stiffened, ankylosed, and deformed through the gouty deposit and the subsequent inflammation. Frequently chalky concretions are visible around the joints chiefly of the hands. They are also not infrequently present upon the helix of the ear and sometimes upon the eyelids. These nodules may make their exit through ulceration of the overlying skin.

The description just given is that of articular or regular gout. There is little difficulty in diagnosing the disease when it appears in this frank form. Frequently, however, it assumes various guises, implicating the heart, kidneys, nerves, respiratory or digestive tract. Such manifestations, which are termed irregular gout, are common in those who inherit the gouty diathesis.

Disorders of the stomach and intestines are commonly observed in gout. The most prominent symptoms are epigastric pain, a sense of distention after eating, acid eructations, and frequently loss of appetite amounting in some cases to actual disgust for food. Occasionally there are violent cramps, vomiting, nausea, and diarrhœa. It is manifest that there is nothing distinctive about these symptoms. They are evidence of the existence of one or another form of dyspepsia. Clinical observers, however, have noted that many of these patients are, in advanced life, attacked with articular gout, the gastric and intestinal symptoms then suddenly disappearing. We have in this proof that the digestive and the articular disorders are simply interchangeable phases of the same diseased process.

The heart and vascular system are often disturbed in gout. Increase of uric acid in the blood is apt to produce high arterial tension, hypertrophy of the heart, and later arterio-sclerosis and angina pectoris.

Chronic bronchitis and asthma have long been known to be frequently associated with gout.

Eczema, psoriasis, and certain other skin affections are commonly observed in patients with the uric acid habit.

It is but natural that the urinary tract, being the chief eliminatory avenue, should suffer in this disease. In many cases the irritation of the kidney by the excess of urates leads insidiously to chronic interstitial nephritis known as the gouty kidney. Deposition of the urates in the kidneys may lead to the formation of calculi, and it is here to be noted that the uric acid stone is by far the most common variety of both the renal and the vesical calculus. Painful and frequent urination occurs in a certain number of lithæmic sufferers, and burning at the neck of the bladder and at the urinary meatus are among the commonplaces of practice.

The nervous system does not remain free from the encroachments of the uric acid disorder. Headache, especially migraine, not infrequently alternates with joint pains. Haig was able to produce attacks of migraine in himself at will, by resort to an exclusive meat diet for several days. Mental disturbances, such as depression of spirits, melancholia, hypochondria, and excessive irritability, are not among the rarities of experience. Neuralgias of the greatest obstinacy and severity are unfortunately common consequences of gout. They resemble the evolution of articular gout in their paroxysmal character and their tendency to recurrence. First in frequency is sciatica, which may occur as a simple neuralgia or an uncompromising neuritis. The next most frequent nerve attacked is the trifacial and its various branches.

In addition to the diseases above mentioned, gout has numerous morbid affinities. That gall-stone bears a definite relation to gout is proved by the frequency with which this condition is found in individuals of gouty ancestry. An intimate relation between gout and diabetes long ago attracted the attention of physicians. Both diseases occur in florid, corpulent subjects who are great eaters and of inactive habits. Obesity is another morbid condition that is closely affiliated with gout. The children of gouty patients are apt to be fat, and, on the other hand, the offspring of the obese are prone to develop gout. Excessive corpulence is by no means always due to gormandism, but is dependent upon certain deeply rooted nutritional errors peculiar to some constitutions.

There is another disease that I desire to touch upon. It is one, however, with which you are all much more familiar than I am. For my information upon the subject I am entirely indebted to the valuable articles written by dental practitioners of this city. The affection is pyorrhœa alveolaris of constitutional origin. And at this juncture I desire to congratulate you all upon the masterly scientific work that has been done in connection with this disease. Its recognition as one of the manifestations of gout reflects the greatest credit upon the dental profession, and particularly upon those members of the profession of this city who have by their close observation and clear judgment divined its true nature. Such work as this will force the profession at large to recognize dental medicine as a legitimate branch of the great healing art. I am sorry to say that in medical works on gout there is a surprising absence of allusion to this form of the disease. Occurring, as I understand it frequently does, before more striking evidences of gout are present, it constitutes a most valuable index of the underlying blood dyscrasia. In this manner its importance as a diagnostic clue in the detection of latent phases of this disease cannot be over-estimated. From a pathological point of view pyorrhœa alveolaris is strikingly analogous to articular gout. In an attack of gout there occurs a deposit of uratic salts in the cartilage and synovial membranes of the joint, followed by inflammation and subsequent degenerative changes. In pyorrhœa the joint affected is the dento-alveolar articulation, with the precipitation of the uratic crystals in the pericemental membrane. Inflammation then occurs, the subsequent changes being only modified by the different environment. There is another dental condition that sometimes occurs in gout. Flint says that Graves calls attention to the habit of grinding teeth as peculiar to cases of gout. The habit proceeds from an uneasy sensation in the teeth, which is in this way momentarily relieved. This symptom occurs more frequently in adults, and the teeth may be considerably worn down by attrition.

*Treatment.*—As far as gout is concerned, it is unfortunate that individuals have no choice in the selection of their parents, for the sins of gouty fathers are certainly visited upon their children. Prophylaxis is an all-important feature of the treatment, and the key-note of prevention is moderation in eating and drinking. The Germans may laugh, as they do, at the water-drinking Americans, and cynically remark that water is fit only for washing; we may answer that it is the best means of human irrigation, and that water-drinkers seldom acquire gout. Adherence to the use of nature's beverages, milk and water, would save many devotees of Bacchus from chronic invalidism and untimely death. As has been already stated, the least injurious of alcoholic beverages are claret, Rhine wines, and brandy and whiskey. Used in moderation these are not productive of great harm. Both a quantitative and qualitative regulation of diet is necessary in the treatment of gout. It is not necessary to absolutely interdict the use of meat, but the quantity should be restricted to a small portion once a day. Fish may often be substituted for red meat with advantage. The vegetables which are to be avoided by gouty patients are asparagus, tomatoes, and green peas. Rhubarb had better also be eschewed. Luff has experimentally proved that the solubility of the biurate of sodium is markedly increased by the mineral constituents of certain vegetables, particularly spinach, Brussels sprouts, French beans, winter cabbage, turnips, and celery. Patients should be encouraged to freely partake of these articles. The starchy and saccharine foods are often poorly borne by the gouty, so it is advisable that these classes of food-stuffs should be used in moderation.

The excessive use of common table-salt lessens the solubility of sodium biurate, and it should therefore be employed as sparingly as is compatible with the palatability of the food eaten. The ordinary fruits may be added to the dietary without disadvantage.

Proper exercise graduated to the physical make-up of the patient is essential. Even though gout be explained upon a theory other than suboxidation, it is evident that by stimulating elimination through the sweat-glands much of the burden of blood purification is taken from the kidneys. Gout is particularly apt to develop in those who are physically indolent, who lead sedentary lives, and who do not properly get rid of the waste products in the system.

*Medicinal Treatment.*—Mineral waters have been so largely used in the treatment of the uric acid diathesis that the industry of manufacturing natural mineral waters has been stimulated to a profitable degree. It appears from recent research that those waters containing considerable proportions of the sodium salts had better not be employed. The potassium and lithium waters in addition to being alkaline, have a distinct diuretic influence which is most desirable. Pure unmedicated spring water is in itself capable of accomplishing great good. The total amount of fluid taken in the course of twenty-four hours should not be less than a gallon, most of which should be imbibed between the meals.

Among drugs the oldest and most efficient is colchicum. It is believed to have been used by the Greeks and Arabians under the name of hermodactylus. Most of the patent preparations for gout contain this as their essential ingredient. How colchicum does good in gout is not definitely known, but many years of experience have testified to its unimpeachable efficiency. In acute attacks of gout the wine of the root of colchicum may be administered in ten- to twenty-minim doses every few hours until nausea or slight purging is produced. In chronic gout ten drops three times a day is the average dosage. The salicylates are frequently employed, but Luff believes them to do harm.

Effervescent tablets of citrate of lithia are now extensively employed, and by their use one may manufacture in a few minutes an excellent and inexpensive lithia water. Various combinations of remedies have been from time to time recommended, but the necessary brevity of this paper has prevented more than a general outline of the therapeutic measures to be employed.

